# Mitochondrial Open Reading Frame of the 12S rRNA Type-c: Potential Therapeutic Candidate in Retinal Diseases

**DOI:** 10.3390/antiox12020518

**Published:** 2023-02-18

**Authors:** Zahra Mohtashami, Mithalesh Kumar Singh, Farid Thomaz Neto, Nasim Salimiaghdam, Hossein Hasanpour, M. Cristina Kenney

**Affiliations:** 1 Department of Ophthalmology, Gavin Herbert Eye Institute, University of California Irvine, Irvine, CA 92697, USA; 2 Department of Pathology and Laboratory Medicine, University of California Irvine, Irvine, CA 92697, USA

**Keywords:** MOTS-c, mitochondrial dysfunction, glaucoma, diabetic retinopathy, age-related macular degeneration

## Abstract

Mitochondrial open reading frame of the 12S rRNA type-c (MOTS-c) is the most unearthed peptide encoded by mitochondrial DNA (mtDNA). It is an important regulator of the nuclear genome during times of stress because it promotes an adaptive stress response to maintain cellular homeostasis. Identifying MOTS-c specific binding partners may aid in deciphering the complex web of mitochondrial and nuclear-encoded signals. Mitochondrial damage and dysfunction have been linked to aging and the accelerated cell death associated with many types of retinal degenerations. Furthermore, research on MOTS-c ability to revive oxidatively stressed RPE cells has revealed a significant protective role for the molecule. Evidence suggests that senescent cells play a role in the development of age-related retinal disorders. This review examines the links between MOTS-c, mitochondria, and age-related diseases of the retina. Moreover, the untapped potential of MOTS-c as a treatment for glaucoma, diabetic retinopathy, and age-related macular degeneration is reviewed.

## 1. Introduction

Mitochondrial DNA (mtDNA) encodes for numerous small polypeptides, termed mitochondrial-derived peptide (MDP), from their Short Open Reading Frame regions. These MDPs include humanin (HN), six small-humanin like peptides (SHLP1-6), and MOTS-c [1]. MDPs have been shown to reverse insulin resistance, slow the aging process, and reduce inflammation in rodent studies [2,3,4]. They are known to provide a wide array of protective effects, including neuroprotective, cytoprotective, antioxidant, anti-inflammatory, and metabolic properties which would be valuable in the treatment of retinal diseases [5,6]. Therefore, it is not surprising that all MDPs discovered to date have either altered expression under age-related pathological conditions or can ameliorate symptoms when administered exogenously. 

The MDP that has been evaluated the most is HN, which is cytoprotective in numerous neurodegenerative diseases and shows anti-apoptotic activity in cybrid cell lines carrying mitochondria from patients with age-related macular degeneration (AMD) [5]. Importantly, Nashine et al., showed that damaged mitochondria in the AMD patients be rescued by treatment with Humanin-G, a cytoprotective peptide that increased cellular longevity [7]. The rate of damage to mtDNA is higher than that to nuclear DNA in studies with human RPE cells exposed to oxidative stress [8]. Six other peptides, designated SHLP1-6, with lengths ranging from 20 to 38 amino acids, are also located on the 16S rRNA, alongside humanin. SHLP6 induced cell death, while SHLP2 and SHLP3 prevented cell death. SHLP2 acted similarly to HN in protecting primary mouse cortical neurons from toxicity caused by beta-amyloid (Aβ_1-42_). The differentiation of insulin-dependent adipocytes was hastened by SHLP2 and SHLP3 [1].

Mitochondria are found in the retinal pigment epithelial (RPE) basal region, near the photoreceptors (PRs). In the inner retina, however, mitochondria are predominantly concentrated in the unmyelinated proximal axons of retinal ganglion cells (RGCs), which transmit visual information to the brain [9]. Over time, oxidative damage caused by mtDNA instability leads to more damage to the mitochondria, which is known to be a major cause of age-related eye disorders [10]. 

Reactive oxygen species (ROS) and reactive nitrogen species (RNS) are two types of free radicals that can be produced as metabolic byproducts of oxidation-reduction reactions in living cells [11]. Unstable molecules with an odd number of unpaired electrons are known as free radicals. It is important to note that both endogenous and exogenous substances can contribute to ROS production [11]. High ROS can react with a wide variety of biomolecules (such as proteins, lipid membranes, and DNA) and causing harm to cells. High level of ROS and free radicals, or low levels of antioxidants, characterize oxidative stress [12,13]. Dynamic redox balance between ROS production and free radical scavenging may be achieved under physiological conditions. Massive accumulation of ROS occurs in response to different overabundant stimuli (both endogenous and exogenous). This may cause oxidative stress in the affected tissues [14].

The review looks at the potential beneficial effect of MOTS-c on age-related retinal diseases, such as glaucoma, diabetic retinopathy (DR), and age-related macular degeneration AMD, as well as the connection between mitochondria and these diseases.

## 2. Role of Mitochondria in the Retina

Among all human tissues, the retina uses the most oxygen [15,16,17]. Among all forms of central nervous tissue, the inner retinal neurons have the highest metabolic rate, and the photoreceptors’ oxygen consumption rate is even higher. The ionic pumps that power the “dark current” are powered by the ATP produced by the mitochondria found in the inner segments of PR cells [15,17,18]. The retina receives a lot of direct exposure to visible light, and it also has a lot of photosensitizers [19]. Normally, oxidative damage is kept to a minimum by the body’s own antioxidants and repair mechanisms [20]. 

In retinal diseases, there is a slow shift toward exacerbation of these changes [20,21]. The neural retina and retinal pigment epithelium rely on mitochondria for their metabolic maintenance. As a matter of fact, photoreceptors have low reserves of mitochondrial energy and are, thus, especially vulnerable to disturbances in energy homeostasis [22]. It is, thus, not surprising that the most common retinal disorders, i.e., glaucoma, AMD, and DR, show mitochondrial dysfunction [23]. Oxidative stress and apoptosis are involved in the final pathological stages of retinal diseases, such as photoreceptor degeneration, diabetic retinopathy, and retinal ganglion cell injury [24]. The 4977-base pair (bp) common deletion (mtDNA4977) is a marker of mtDNA damage caused by oxidative stress. This deletion affects more than a quarter of the mitochondrial genome and disrupts three complexes of genes that code for proteins involved in oxidative phosphorylation (OXPHOS) [25,26]. OXPHOS, a main nut in energy production, is comprised of four respiratory chain complexes included in electron transport, generation of the proton gradient in the mitochondrial intermembrane space, and ATP synthesis. Moreover, OXPHOS has a role in apoptosis and radical production.

**Figure 1:**
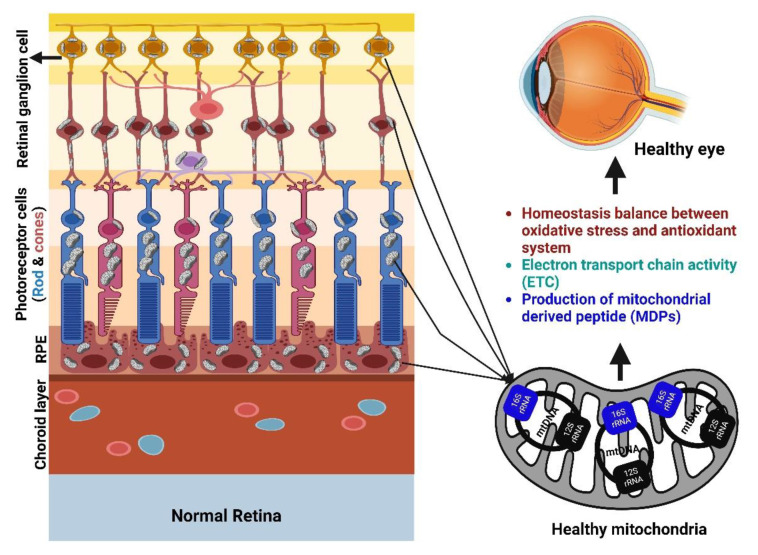
Mitochondria’s biological role in the retina of a healthy eye. Created with Biorender.com.

Dysregulated mitochondria result in significantly less energy production and enhanced apoptosis, and severely affect tissue homeostasis [27]. Defects of the mitochondria have been implicated in aging and the pathogenesis of numerous retinal degenerations [10,20]. Mutations in mtDNA can cause lower ATP synthesis, increased generation of toxic ROS, and significant decline in mitochondrial functions, all of which are universal features of normal ageing. Mitochondrial disorders mainly affect organs and cells that are highly metabolic and depend on aerobic respiration including: nervous system, heart, skeletal muscles, renal tubules, endocrine glands, ears, and in the eyes (Figure 1). Mitochondrial dysfunction is a hallmark of each of these diseases, and oxidative stress is a key contributor to this dysfunction when the cell’s antioxidant defenses are outmatched by ROS [28,29,30].

## 3. MOTS-c and Its Role in Mitochondrial Biogenesis and Mitophagy

Of the known native MDPs, MOTS-c has most consistently been reported to have metaboloprotective properties in multiple models of metabolic dysfunction [31]. The MDPs have an important function as mitochondrial retrograde signaling molecules that modulate the status of mitochondrial health. The mitochondrially encoded 12S rRNA region encodes for MOTS-c, a 16-amino-acid peptide with the sequence of MRWQEMGYFYPRKLR [31]. 

Translation of MOTS-c must take place in the cytoplasm using the universal genetic code rather than the mitochondria-specific genetic code due to the presence of tandem start and stop codons in the mitochondrial translation [32]. Loss of MOTS-c over time after selective depletion of mitochondrial RNA suggests that the MOTS-c transcript is likely generated in the mitochondria before being exported to the cytosol by an unknown mechanism [32]. Under normal conditions, MOTS-c is found localized to mitochondria [32,33]. It has already been established that some mitochondrial proteins are translated and translocated into the mitochondria after being produced by mitochondrial associated cytoplasmic ribosomes [34,35]. 

By inhibiting respiration and increasing glucose uptake, MOTS-c is the mitochondrial signal that promotes cellular health. According to the “Crabtree effect”, glucose can suppress cellular respiration and OXPHOS [36]. Glucose ingested in response to MOTS-c was diverted from glycolysis and instead metabolized via the anabolic pentose phosphate pathway (PPP), which supplies carbon sources for the synthesis of purines [32]. Furthermore, MOTS-c increased the level of antioxidant intermediate and decreased intracellular levels of essential and non-essential fatty acids, indicating improved lipid utilization [32]. Carnitine shuttles are responsible for transporting activated fatty acids into the mitochondria for β-oxidation.

MOTS-c is strongly linked to amino acid, carbohydrate, and lipid metabolism and serves as a key regulator for energy balance. It is encoded in mammalian cells by mitochondrial DNA and translocates to the nucleus in response to stress, where it promotes increased production of reactive oxygen species (ROS). MOTS-c nuclear translocation requires 5′-adenosine monophosphate-activated protein kinase (AMPK). By blocking both the folate cycle and de novo purine biosynthesis, MOTS-c activates AMPK and leads to an increase in 5-aminomidazole-4-carboxamide ribonucleotide (AICAR), a known AMPK activator [4].

**Figure 2:**
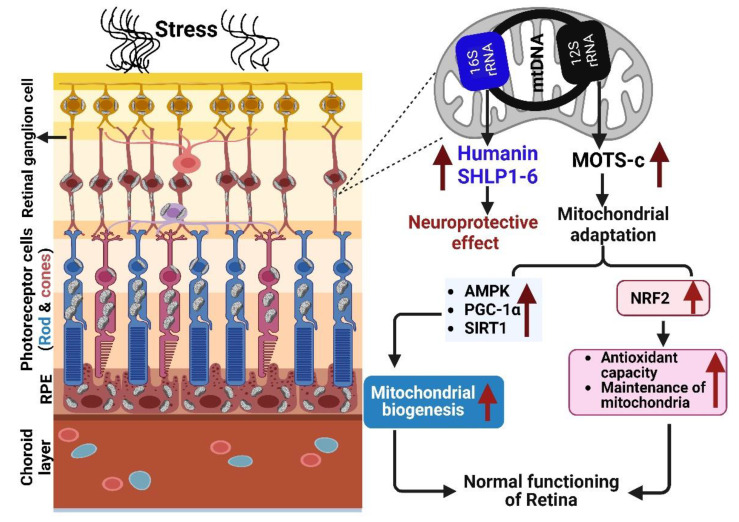
The mechanism of mitochondrially derived peptides, including MOTS-c, humanin, and SHLP1-6, in the retina. Red arrow indicates upregulation/increased expression. Created with Biorender.com.

MOTS-c regulates the nuclear genome and maintains cellular homeostasis in stress conditions by providing an adaptive stress response [33,37]. Furthermore MOTS-c potentiates Nuclear Factor Erythroid 2-Related Factor 2 (NFE2L2/NRF2) as antioxidant response element (ARE) transcription factors [3,38,39], which are stress-responsive factors that protect cells from oxidative stress [40]. In RPE, MOTS-c is found to be mainly in the perinuclear region and cytoplasm [5]. It co-localizes largely with mitochondria in unstressed RPE cells, and less in the nuclei (Figure 2). However, in response to metabolic or oxidative stress, MOTS-c rapidly translocates into the nucleus [4,5]. MOTS-c triggers the activation of AMP- activated protein kinase (AMPK) and accumulation of 5-aminoImdazole-4-carboxAmide ribonucleotide (AICAR), a known AMPK activator, by inhibiting the folate cycle and de novo purine biosynthesis [41,42]. 

The nuclear factor kappa-B (NF-κB) is one of the most well-known downstream targets of AMPK signaling in the inflammation process. Moreover, MOTS-c elevates nicotinamide adenine dinucleotide+ (NAD+), a potent Sirtuin (SIRT) activator, which plays a key role in energy metabolism, cell survival, and aging. SIRT1 deacetylates (at p65 subunit) and reduces NF-kB signaling that protects neurons from amyloid beta induced toxicity in microglia [43]. Under energetic stress condition, AMPK and SIRT1 negatively regulate Mammalian target of rapamycin Complex 1 (mTORC1) to conserve energy for cell survival [44,45,46]. mTOR is an evolutionarily conserved serine/threonine protein kinase that regulates multiple cellular processes such as cell growth, cell cycle, cell survival, and autophagy [47]. Recent studies show that mTOR inhibition mitigates oxidative stress and promote mitochondrial function to improve lifespan [48,49]. 

MOTS-c is a key regulator of the signaling pathways that initiate mitochondrial biogenesis, including the proliferator-activated receptor Gamma Coactivator 1 alpha (PGC-1α). PGC-1α translocates to the nucleus upon activation and acts as a coactivator of transcription factors such as NRF-1 and NRF-2, as well as mitochondrial transcription factor A (TFAM), which are involved in OXPHOS and mtDNA replication [50,51,52]. PGC-1α protects neural cells in culture from oxidative stress and delays the onset of conditions associated with aging [53]. A recent study using human ARPE19 cells reported that elevated PGC-1α is essential for maintaining normal autophagic flux [54]. In a rodent glaucoma model, an AMD model, and cultured RGC-5 cells, activating AMPK/PGC-1α signaling decreased cell apoptosis through its activation by different compounds [55]. 

MOTS-c regulates Sirtuins expression. Sirtuins belong to a conserved family of nicotinamide adenine dinucleotide- (NAD-)dependent protein deacylases [56,57] that modulate metabolism with autophagy and mitophagy. They are associated with aging, metabolism, DNA repair, and prevention of age-related ocular diseases [58]. SIRT1 has been extensively studied due to its ability to deacetylate PGC-1𝛼, NF-𝜅B, and p53 [59]. 

It has been reported that MOTS-c acts as a cytoprotective agent in various cell types. Protective effects against glucose/serum restriction were significantly enhanced in HEK293 cells that stably overexpressed wild-type MOTS-c compared to cells transfected with empty vector control [33]. MOTS-c is crucial in preventing inflammation caused by lipopolysaccharide-induced acute lung injury [60]. It has been called a mitochondrial-encoded hormone or a mitochondrial cytokine (mitokine) [46,61,62,63] due to its dual roles as an intracellular and endocrine factor. Multiple avenues, such as cellular uptake, are being explored in order to better understand the molecular details of MOTS-function as an endocrine factor.

MOTS-c has consistently been demonstrated to have metabolism protective properties in multiple models of metabolic dysfunction and its circulating level in humans are reportedly reduced by aging, obesity [32], insulin resistance [32], diabetes [6], endothelial dysfunction [64], and chronic kidney disease [65]. Indeed, clinical trials on MOTS-c and a MOTS-c analogues are under way, with potential indications for coronary artery disease in patients with type 2 diabetes (NCT04027712) and nonalcoholic hepatic steatosis and obesity (NCT03998514) [31].

## 4. Age-Related Retinal Diseases

**Figure 3:**
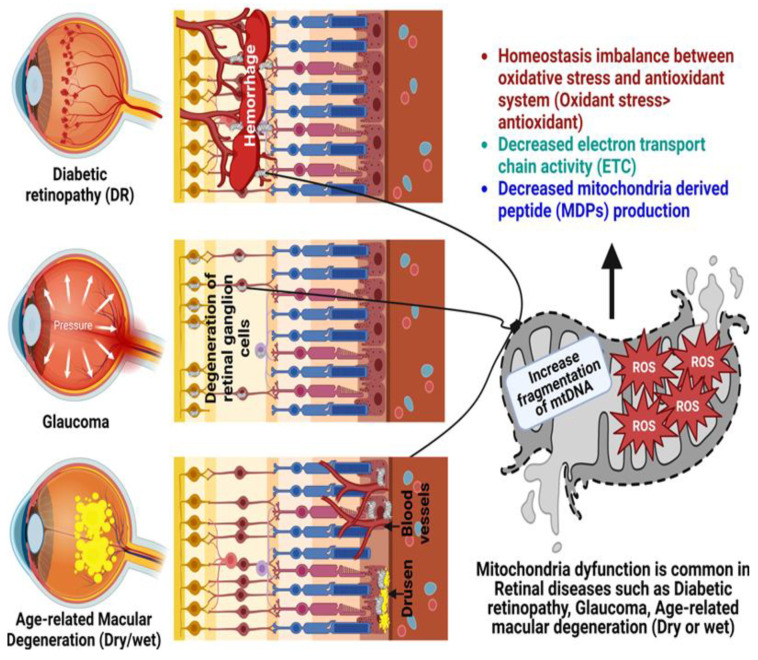
Role of mitochondrial dysfunction in the pathogenesis of glaucoma, diabetic retinopathy (DR), and age-related macular degeneration (AMD). Created with Biorender.com.

Metabolic dysfunction in neuronal cells is associated with the development of age-related neurodegenerative diseases. The net result of metabolic dysfunction includes reduced bioenergetics, increased generation of mitochondrial ROS, mitochondrial dysfunction, and cell death [14,66]. With aging, the human retina undergoes various structural and physiologic changes [67]. Aging has been associated with fewer retinal neurons (Rod PRs, RGCs, Rod bipolar cells), along with numerous age-related alterations, such as higher rate of mtDNA mutations and decreased density of cells and synapses [68,69]. mtDNA becomes damaged with age and oxidative stress, and the absence of good repair mechanisms leads to accumulative mitochondrial dysfunction, which is recognized as a crucial pathogenic element in age-related ophthalmic diseases (Figure 3), such as glaucoma, DR, and AMD [70].

### 4.1. Glaucoma

Progressive loss of vision is the outcome of glaucoma, a neurodegenerative disease of the optic nerve characterized by the accelerated death of RGCs and their axons. Since RGCs are located in the optic nerve, they are especially vulnerable to mitochondrial respiratory capacity damages [71,72]. As the world’s population ages, experts predict that glaucoma’s prevalence will rise, ultimately reaching 111.8 million people by the year 2040 [73]. In common with other neurodegenerative disorders, increasing age is a major risk factor for the prevalence and incidence of glaucoma. The causes of RGC degeneration in glaucoma are probably multifactorial [74]. The link between increased age and prevalence of glaucoma suggests that aging may make the optic nerve more susceptible to various stressors, which eventually results in RGC death and optic nerve degeneration [75]. In the early stages of glaucoma development, oxidative stress contributes to cellular damage in the trabecular meshwork, changes in the homeostasis of nitric oxide and endothelin, and finally, a role in cellular death in the ganglion cells [76]. An association between glaucoma and mitochondrial dysfunction has been suggested in a recent clinical study, where a 21% reduction in mitochondrial respiratory function and an increase in mtDNA mutations were observed in peripheral blood of patients with primary open-angle glaucoma [77,78].

Mitochondrial dysfunction is present in glaucoma animal models prior to RGC death [79,80], suggesting a primary effect for mitochondrial abnormalities in glaucoma onset and subsequent progressive loss of vision. In animals, elevated Intraocular Pressure (IOP), a primary feature of glaucoma, reduces antioxidant defenses and increases oxidative stress, which may predispose RGC to apoptosis and degeneration [81,82]. Blocking ROS can reduce mitochondrial dysfunction, and thus, slow the progression of glaucoma. This is because ROS are byproducts of electron outflow along the electron transport chain during cell respiration [83,84]. 

Exogenous application of ROS triggers RGC apoptosis in vitro via caspase-independent pathways [85], while reduction of ROS generation temporarily protects RGCs from apoptosis [86]. An important transcriptional factor in these events is Nrf2, which influences the expression of a diverse array of antioxidant pathways, such as glutathione, as well as cytoprotective genes. Another important regulator of the antioxidant defense system is PGC-1𝛼, which modulates the antioxidant proteins that are not regulated by Nrf2 [87,88]. PGC-1α has been shown to regulate astrocyte activities and RGC homeostasis [89]. Moreover, SIRT1 overexpression in mice showed significantly higher RGC number compared to wild type [90]. Preliminary research shows that resveratrol (a SIRT1 activator) treatment of mice at 250 mg/kg mitigates RGC loss and maintains pupillary light responses. SIRT1 controls vascular endothelial growth factor-A (VEGF-A) by activating hypoxia-inducible factor-2 alpha (HIF-2α), as was noticed in a study conducted on hypoxic choroidal endothelial cells by Balaiya et al. SIRT1 deacetylates (at p65 subunit) and reduces NF-kB signaling that protects neurons from amyloid beta induced toxicity in microglia [90,91,92]. It has been suggested that as a potent SIRT1 activator, MOTS-c would have the same effect as Resveratrol on RGCs [93]. Additionally, the MOTS-c peptide could induce antioxidant and cytoprotective genes expression by PGC-1𝛼 activation and NRF2 upregulation, which maybe potentially useful for glaucoma treatment. MOTS-c can target mitochondria adaptation to restore energy production in cells with impaired OXPHOS, and therefore, may provide a potential therapeutic approach in glaucoma [94].

### 4.2. Diabetic Retinopathy

Photoreceptors, bipolar, horizontal, amacrine, and ganglion neurons, Müller cells, astrocytes, microglia, and pigment epithelial cells are all negatively impacted by diabetes [95,96]. Different racial populations (mtDNA haplogroups) have different susceptibilities to develop diabetes [97,98]. DR, the most prevalent microvascular complication of diabetes, occurs gradually within 15 years of diagnosis in as many as 50% of type I diabetics and 10% of the type II diabetic patients [99,100]. However, hyperglycemia is the primary factor in the onset of DR in people with diabetes [101]. ROS production, including superoxide and hydrogen peroxide, is increased in retinal tissues due to hyperglycemia.

Retinal neurodegeneration is an early event during the progression of DR. The pathogenesis of DR involves progressive dysfunction of retinal mitochondria in the setting of hyperglycemia, with mtDNA damage and accelerated apoptosis occurring in retinal capillary cells [102]. In retinal tissues, hyperglycemia upregulates production of ROS, such as superoxide and hydrogen peroxide [85,103]. The cascade of mitochondrial fragmentation, DNA damage, increased membrane potential heterogeneity, decreased oxygen consumption, and cytochrome c release is triggered by oxidative stress. By releasing cytochrome c, mitochondria initiate the caspase cascade, thereby committing the cell to apoptosis [104]. Oxidative stress also triggers activation of NF-κB, which initiates the release of pro-inflammatory cytokines, such as tumor necrosis factor alpha (TNF-α), interleukin 6 (IL-6), IL-8, and IL-1β. Moreover, their expression levels are correlated with the severity of DR [105,106]. 

Since the disease pathogenesis is partially attributed to mitochondrial dysfunction, treatment options should also consider restoring normal mitochondrial function by lowering oxidative stress [107]. Thus, therapies that target multiple steps of oxidative stress and mitochondrial damage should provide a hope for the prevention of this multifactorial blinding complication of diabetes [108,109,110]. The development of retinopathy, on the other hand, has been prevented in animal models of diabetes thanks to antioxidant supplementation and overexpression of mitochondrial antioxidant enzymes [111]. The recognition of MOTS-c and its inverse relationship to age and hemoglobin A1c (HbA1c) [4,112] provide further evidence for the importance of MDP in insulin sensitivity. Investigations by Ramanjaneya et al. showed that subjects with type II diabetes had significantly lower levels of circulating MOTS-c than controls [113]. 

### 4.3. Age-Related Macular Degeneration

AMD is a developing neurodegenerative disease of the central macular region involving PRs, RPE, Bruch’s membrane (BrM), and the choroid [114,115]. AMD affects approximately 20–30% of people over 75 years in the developed world and it is estimated to be increased to 288 million persons by 2040 [116]. Vascular inflammation and dysregulation, mitochondrial damage and accumulation of ROS, and RPE cell senescence are the molecular underpinnings of the transition from normal aging processes to pathological AMD [117]. RPE dysfunction plays a crucial role in pathophysiology of AMD [118] and oxidative stress is a key factor for triggering RPE degeneration [119]. 

The high metabolic activity of RPE cells is due to their numerous mitochondria. By utilizing OXPHOS, mitochondria are the body’s primary source of energy production. Reduced energy production and increased apoptosis are both consequences of mitochondrial dysregulation, which is thought to be a root cause of AMD [120,121,122]. These alterations lead to a decline in bioenergetics, an uptick in mitochondrial ROS production, mitochondrial dysfunction, and ultimately cell death. RPE cells isolated from AMD patients were found to be particularly vulnerable to mtDNA damage [5,123,124,125]. 

Two central elements of RPE damage are NRF-2/ARE and PGC-1𝛼, which undergo downregulation and are associated with increased oxidative stress, lipofuscin accumulation, and mitochondrial damage involved in AMD pathophysiology [126,127]. In patients with AMD, plasma levels of complement regulatory proteins Factor H and Factor I (encoded by the CFH and CFI genes, respectively) as well as some inflammatory cytokines (IL-6 and TNF-α) are elevated [128,129]. 

There is solid evidence that dysfunction of the metabolic ecosystem leads to the retinal degeneration associated with AMD. In a large study on dysregulated metabolic pathways in AMD, Golestaneh et al. reported downregulated AMPK/SIRT1 and PGC-1α pathways along with overactive mTOR expression contributed to the underlying disease mechanisms in AMD RPE. These events could directly affect mitochondrial metabolism and biogenesis [130]. Based on these studies, it is reasonable to speculate that pathways related to energy metabolism, mitochondrial biogenesis, and oxidative stress may be ideal targets to treat AMD. Activation of AMPK promotes downstream energy producing pathways, including glucose metabolism, mitochondrial function, and autophagy. This makes AMPK an ideal target for diseases such as AMD. 

It has been suggested that mtDNA fragmentation and MDPs may play a role in the pathology of AMD, and this is supported by other studies. The mtDNA damage in AMD patients was found to be increased by 350% and localized to specific regions of the mitochondrial genome, including the 16S and 12S ribosomal RNA genes and eight of the mitochondrial genome’s 22 tRNA genes [5]. Damage to 16S rRNA or 12S rRNA could result in dysregulated production of MDPs. HN and SHLPs, provide cytoprotective functions in RPE cells and ocular diseases and may be considered as potential therapeutic targets for AMD [7,131,132]. MOTS-c is primarily expressed in the perinuclear region and the cytoplasm of RPE. In unstressed RPE cells, MOTS-c co-localized primarily with mitochondria, with negligible localization in the nucleus [5,33]. Serum-starved RPE cells cotreated 24 h with tert-Butyl Hydroperoxide (tBH) (150 µM) and increasing doses of MOTS-c (1 µg to 10 µg) demonstrated elevated protection of RPE cells, with the highest dose providing the most protection.

## 5. Future Directions

Mutations, fragmentation, and disruption of mtDNA accumulate with age, causing progressive mitochondrial damage in the human retina as a result of the aging process. Age-related retinal diseases, such as diabetic retinopathy, age-related macular degeneration, and glaucoma, all have these occurrences as important pathogenic factors. Eventually, more and more people will be affected by age-related ocular diseases. It is estimated that by 2050, 470 million people will have some degree of visual impairment and 61 million will have total vision loss [133]. The healthcare system will be taxed by the needs of the millions of people who are blind or visually impaired, as well as their family members and other caregivers. In the medical field, there is often a dearth of effective treatments for patients who are already in the late stages of a disease. Since the adaptive phase and the early pathology phase in retinal diseases are reversible and the most effective phases, respectively, it is crucial to establish methods of early detection of retinal diseases and monitoring of disease progression and treatment efficacy [134]. 

The integrity of the MDPs relies on the integrity of the mtDNA, as they are encoded directly from the 16S rRNA and 12S rRNA regions of the mtDNA. There is a risk that MDPs will lose their efficacy if the mtDNA is degraded or mutated. MDPs are crucial mitochondrial retrograde signals that directly reflect the state of mitochondrial health. Because they control mitochondrial metabolism and processes like aging, inflammation, and insulin resistance, they may be useful in treating retinal diseases. In times of adversity, MOTS-c plays a crucial role as a regulator of the nuclear genome, encouraging the right kind of stress response to help keep things steady in the cell. Possible AMPK-regulated partners for MOTS-c shuttle. Even though the connection with AMPK is obvious, its role as a nuclear gatekeeper for MOTS-c is fascinating in and of itself. Keep in mind that although AMPK is well-known as a serine/threonine kinase, the absence of MOTS-c containing residues in its sequence indicates the involvement of intermediates. Identifying MOTS-c specific binding partners may help in deciphering the complex web of mitochondrial and nuclear-encoded signals. In addition, research on MOTS-c potential to revive oxidatively stressed RPE cells has revealed a significant protective role for this molecule. Evidence suggests that senescent cells play a role in the development of age-related retinal disorders; it seems plausible that HN and related compounds could one day be used as senolytic medications. More research is needed in this emerging field before it can provide conclusive answers, especially concerning the in vivo MOTS-c functions.

## 6. Conclusions

**Figure 4:**
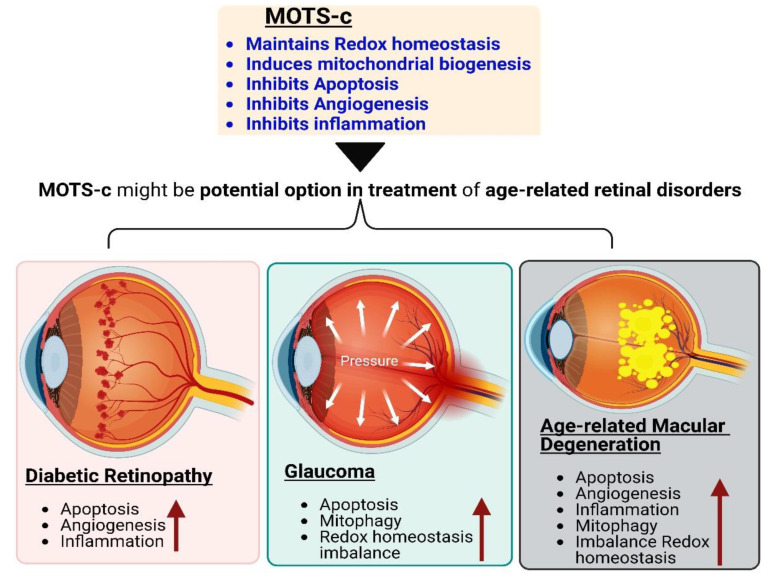
MOTS-potential c’s benefits make it an ideal candidate for treating diabetic retinopathy, glaucoma, and age-related macular degeneration.

Even though mitochondria are at the heart of cellular signaling, MDPs, specifically MOTS-c, would provide promising results in the treatment of retinal disorders caused by mitochondrial dysfunction (Figure 4). The goal of this review was to show the untapped potential of MOTS-c as a therapeutic option in the treatment of age-related retinal diseases.

## Data Availability

Not applicable.
